# The role of family history of Cancer in Oral Cavity Cancer

**DOI:** 10.1186/s13005-021-00298-8

**Published:** 2021-11-22

**Authors:** Paolo Junior Fantozzi, Roxanne Bavarian, Ibon Tamayo, Marie-Abele Bind, Sook-Bin Woo, Alessandro Villa

**Affiliations:** 1 Division of Oral Medicine and Dentistry, Brigham and Women’s Hospital, 75 Francis St., Boston, MA 02115 USA; 2 Division of Oral Medicine and Dentistry, Brigham and Women’s Hospital, Boston, MA USA; 3 Department of Oral Medicine, Infection, and Immunity, Harvard School of Dental Medicine, Boston, MA USA; 4 Department of Statistics, Faculty of Arts and Sciences, Harvard University, Cambridge, MA USA; 5 Department of Orofacial Sciences, University of California San Francisco, San Francisco, CA USA

**Keywords:** Oral cancer, Oral cavity, Risk factors, Family history, Immunosuppression, Cancer

## Abstract

**Objectives:**

Oral and oropharyngeal squamous cell carcinoma (SCC) is the 10th most common cancer in the United States (8th in males, 13th in females), with an estimated 54,010 new cases expected in 2021, and is primarily associated with smoked tobacco, heavy alcohol consumption, areca nut use and persistent high-risk human papillomavirus (HPV). Family history of cancer (FHC) and family history of head and neck cancer (FHHNC) have been reported to play an important role in the development of OSCC. We aimed to investigate the role of FHC, FHHNC and personal history of cancer in first/second degree-relatives as co-risk factors for oral cancer.

**Methods:**

This was a retrospective study of patients diagnosed with OSCC at the Division of Oral Medicine and Dentistry at Brigham and Women’s Hospital and at the Division of Head and Neck Oncology at Dana Farber Cancer Institute. Conditional logistic regressions were performed to examine whether OSCC was associated with FHC and FHHNC of FDRs and SDRs, personal history of cancer and secondary risk factors.

**Results:**

Overall, we did not find an association between FHC, FHHNC and OSCC risk, whereas patients with a cancer history in one of their siblings were 1.6-times more likely to present with an OSCC. When secondary risk factors were considered, patients with a history of oral leukoplakia and dysplasia had a 16-times higher risk of having an OSCC.

**Conclusions:**

Our study confirmed that a previous history of oral leukoplakia or dysplasia was an independent risk factor for OSCC. A positive family history of cancer in one or more siblings may be an additional risk factor for OSCC.

## Introduction

Oral and oropharyngeal squamous cell carcinoma (SCC) is the 10th most common cancer in the United States (8th in males, 13th in females), with an estimated 54,010 new cases expected in 2021, a 5-year relative survival rate of 65% and incidence rates more than twice as high in men than in women [1, 2]. Oral squamous cell carcinoma (OSCC) alone, including tongue, mouth, and other oral cavity sites (International Classification of Diseases 10th revision (ICD-10; C00-C08) [3] represents 66% of oral and oropharyngeal cancers and is primarily associated with smoked tobacco, heavy alcohol consumption, areca nut use (mainly in South Asian countries) [4–6], and persistent high-risk human papillomavirus (HPV) infection which, however, only accounts for 3–5% of OSCCs [7, 8]. Other emerging and potential risk factors for OSCC include local and systemic immunosuppression, UV-light exposure (lip cancer only), a low-antioxidant diet, socio-economic status, oral potentially malignant disorders (OPMDs), inheritable cancer syndromes (i.e. Cowden syndrome, Bloom syndrome, dyskeratosis congenita, etc), and familiar genetic alterations [9].

Family history of cancer (FHC) has been reported to play an important role in the development of different cancer types in first-degree-relatives (FDRs, namely parents, siblings and children), with recent studies pointing toward a family history of head and neck cancer (FHHNC), as well as the loss of function TP53 mutations and CDKN2A inactivation, expression of certain proto-oncogenes and frequent copy number alterations [10–13]. The aim of this study was to identify the potential role of FHC, FHHNC and personal history of cancer in FDRs and second-degree relatives (SDRs, namely grandparents, aunts, uncles, cousins, nieces and nephews) in the development of OSCC.

## Methods

### Study design and data description

This was a retrospective case-control study of patients aged greater than or equal to 18 years who were diagnosed with OSCC at the Division of Oral Medicine and Dentistry at Brigham and Women’s Hospital (BWH) and at the Division of Head and Neck Oncology at Dana Farber Cancer Institute (DFCI) from January 2000 through March 2016. Eligible cases included patients with a diagnosis of SCC of the tongue, gingiva, floor of mouth, palate, and oral mucosa (International Classification of Diseases - ICD10-C00-C08). Oropharyngeal SCCs (i.e. oropharynx, base of tongue and tonsils; ICD10-C01.9, C09.0-C09.9 and C10.0-C10.9) were excluded from this analysis given that the majority (79.2%) are HPV-associated and thus a separate etiological entity.

Controls were selected from patients aged ≥18 years seen in the Division of Oral Medicine and Dentistry at BWH between January 2000 and March 2016 for one of the following benign oral conditions: trigeminal neuralgia (ICD10-G50.0), atypical facial pain (ICD10-G50.1), temporomandibular disorder (ICD10-M26.60), burning mouth syndrome (ICD10-K14.6), aphthous stomatitis (ICD10-K12.0), oral fibroma (ICD10-D10.39), geographic tongue (ICD10-K14.1), orofacial granulomatosis (ICD10-K13.4), varix (ICD10-I86.8), and mandibular/maxillary torus (ICD10-M27.8). Exclusion criteria of controls included a current diagnosis of head and neck cancer (ICD10-C00-C14) or a potentially malignant condition such as oral leukoplakia (ICD10-K13.21), oral dysplasia (ICD10-K13.29) and oral submucosal fibrosis (ICD10-K13.5). The study protocol was approved by the Institutional Review Board of the Brigham and Women’s Hospital/Dana Farber Cancer Center.

### Statistical analysis to identify the risk factor for OSCC

Data was collected verbally from prior patient encounters and included patient demographics, medical history, FHC (both of any cancer and of head and neck cancer, separately), and social history. Cases and controls were exact matched by sex, alcohol, and tobacco. Conditional logistic regressions were performed to examine whether OSCC was associated with FHC and FHHNC of FDRs and SDRs, personal history of cancer, history of leukoplakia/dysplasia, as well as history of head and neck radiation therapy. All statistical analyses were carried out using R statistical software (R core team, 2019).

## Results

### Patients characteristics

A total of 607 patients with a diagnosis of OSCC and 396 controls were identified. After removing data with missing values in pertinent categories such as tobacco history, alcohol history, FHC, and FHHNC, 544 cases (59% males, median age: 63 (range: 18–98)) and 359 controls (23% male, median age: 61 (range: 20–96)) remained in the dataset. The majority of the patients were Caucasians (92.7% of cases (*n* = 496), 79.1% of controls (*n* = 284)), males in cases (58.6%) and females in controls (76.9%) with a median age of 63 years (range: 18–98) in the cases and 61 years in the controls (range: 24–96). Amongst the cases, the most common anatomic site for the OSCC was the floor of the mouth (*n* = 158; 29.0%), followed by the gingiva (*n* = 114; 20.9%), and palate (*n* = 77; 14.1%). More than 70% of the cases were ever-tobacco users (*n* = 404; 74.6%), and current alcohol drinkers (*n* = 298; 54.8%), whereas about two-thirds of the controls were never tobacco users (*n* = 164; 45.7%) and half were current alcohol users (*n* = 183; 51.0%) (Table 1).

**Table 1:** Characteristics of patients with OSCC and controls

	Non-matched		Matched	
	Cases ***n*** = 544 n (%)	Controls ***n*** = 359 n (%)	Cases ***n*** = 255 n (%)	Controls ***n*** = 255 n (%)
**Age**				
Median	63 (18–98)	61 (24–96)	64 (18–98)	61 (20–96)
**Gender**				
Male	319 (58.6)	83 (23.1)	82 (32.2)	82 (32.2)
Female	225 (41.4)	276 (76.9)	173 (67.8)	173 (67.8)
**Race**				
White	496 (92.7)	284 (79.1)	230 (90.2)	203 (79.6)
Black	17 (3.2)	17 (4.7)	7 (2.7)	15 (5.9)
Asian/Pacific Islander	12 (2.2)	14 (3.9)	9 (3.5)	11 (4.3)
Hispanic	3 (0.6)	27 (7.5)	0 (0.0)	16 (6.3)
Other	7 (1.3)	2 (0.6)	4 (1.6)	2 (0.8)
**BMI**^a^				
Median (Range)	25 (18.8–36.8)	25.7 (20.1–39.1)	25.1 (18.8–37.3)	26.3 (21.1–39.1)
**OSCC Site**^b^				
Floor of the Mouth (C.04)	158 (29.0)	0 (0.0)	64 (25.1)	0 (0.0)
Gingiva (C.03)	114 (20.9)	0 (0.0)	45 (17.6)	0 (0.0)
Palate (C.05)	77 (14.1)	0 (0.0)	42 (16.4)	0 (0.0)
Mouth, NOS (C.06.9)	71 (13.2)	0 (0.0)	39 (15.3)	0 (0.0)
Buccal Mucosa (C.06)	65 (11.9)	0 (0.0)	34 (13.3)	0 (0.0)
Retromolar area (C.06.2)	45 (8.3)	0 (0.0)	16 (6.3)	0 (0.0)
Tongue (C.02.9)	11 (2.1)	0 (0.0)	14 (5.5)	0 (0.0)
Labial mucosa (C.00.5)	3 (0.5)	0 (0.0)	1 (0.4)	0 (0.0)
**Tobacco History**				
Never	138 (25.4)	230 (64.1)	131 (51.4)	131 (51.4)
Former	293 (53.8)	114 (31.8)	109 (42.7)	109 (42.7)
Current	113 (20.8)	15 (4.1)	15 (5.9)	15 (5.9)
Median Pack/years (Range)	40 (1–160)	10 (1–60)	30 (1–160)	10 (1–60)
**Alcohol consumption**				
Never drinker	147 (27.0)	164 (45.7)	126 (49.4)	126 (49.4)
Former	99 (18.2)	12 (3.3)	9 (3.5)	9 (3.5)
Current	298 (54.8)	183 (51.0)	120 (47.0)	120 (47.0)
Median Drinks/week (Range)	12 (1–105)	3 (1–28)	7 (1–45)	5 (1–28)

^a^*BMI* Body mass index; ^b^International Classification of the Diseases (ICD)-10 codes for each area are included in parentheses

### Family history of cancer and head and neck cancer

After exact matching for sex, alcohol, and tobacco use, 255 cases and 255 controls remained in the dataset; a conditional logistic regression was used to examine whether FHC, or FHHNC in first degree relatives (FDRs; i.e. parents, siblings, children) and second degree relatives (SDRs; i.e. grandparents, aunts, uncles, cousins, nieces, nephews) were associated with OSCC. Overall, 253 patients (49.7%; 128 [50.2%] cases, 125 [49.0%] controls) reported a FHC and 26 patients (5.1%; 15 [57.7%] cases, 11 [42.3%] controls) reported a FHHNC (Table 2). Patients with a history of cancer in one or more siblings were associated with a higher OSCC risk (OR: 1.59; 95% CI: 1.17–2.89; *p* < 0.01). No significant association was found between overall family history of cancer (FDRs and SDRs) and OSCC risk (OR: 0.95; 95% CI: 0.82–1.1; *p* = 0.48), between overall family history of HNC (FDRs and SDRs) and OSCC risk (OR: 1.1: 95% CI: 0.59–2.07; *p* = 0.75), and with history of cancer in children (OR:0.52; 95% CI: 0.42–5.31; *p* = 1.50), SDRs (OR: 0.30; 95% CI: 0.67–1.13; *p* = 0.87) and grandparents (OR: 0.53; 95% CI: 0.27–1.03; *p* = 0.53).

**Table 2:** Risk of OSCC by family history of cancer and head and neck cancer in first- and second-degree relatives after exact matching of cases and controls

	Cases ***n*** = 255	Controls ***n*** = 255	***p***-value	OR	95% CI
**Family history of cancer**^**a**^					
**Overall family**					
Negative	127 (49.8)	130 (51.0)	0.48	0.95	0.82–1.1
Positive	128 (50.2)	125 (49.0)			
1	72 (28,2)	67 (26.3)			
2	31 (12.2)	32 (12.5)			
3	22 (8.6)	14 (5.5)			
> 3	3 (1.2)	12 (4.9)			
**Parents**					
Negative	182 (71.7)	161 (63.4)	< 0.05	0.73	0.55–0.98
Positive	72 (28.3)	90 (36.6)			
One parent	58 (22.8)	72 (28.3)			
Both parents	14 (5.5)	18 (8.3)			
**Siblings**					
Negative	203 (79.9)	221 (87.0)	< 0.01	1.59	1.17–2.89
Positive	51 (20.1)	33 (13.0)			
1	35 (13,8)	29 (11.4)			
2	15 (5.9)	4 (1.6)			
3	1 (0.4)	0			
**Children**					
Negative	248 (97.2)	250 (98.4)	0.52	1.50	0.42–5.31
Positive	7 (2.8)	5 (1.6)			
**Second degree**					
Negative	228 (89.8)	227 (89.4)	0.30	0.87	0.67–1.13
Positive	26 (10.2)	27 (10.6)			
1	16 (6.3)	15 (5.9)			
2	8 (3.1)	5 (2.0)			
> 2	2 (0.8)	7 (2.8)			
**Grandparents**					
Negative	244 (96.1)	237 (93.3)	0.06	0.53	0.27–1.03
Positive	10 (3.9)	17 (6.7)			
1	10 (3.9)	11 (4.3)			
2	0	4 (1.6)			
3	0	2 (0.8)			
**Family history of head and neck cancer**^**a**^					
Negative	240 (94.3)	244 (96.6)	0.75	1.11	0.59–2.07
Positive	15 (5.7)	11 (3.4)			
1	14 (5.3)	10 (3.0)			
> 1	1 (0.4)	1 (0.4)			

^a^numbers do not add to 544 as not all patients reported family history of cancer/head and neck cancer

### Other primary and secondary risk factors for OSCC

A conditional logistic regression was used to examine whether other primary (prior personal cancer history) and secondary risk factors (history of leukoplakia/dysplasia, and personal history of head and neck radiation therapy), were significantly associated with risk of OSCC. Overall, patients with a personal history of oral leukoplakia and dysplasia (*n* = 50; 9.8%) had a 16-fold increased risk of having OSCC (OR:15.6; 95% CI: 4.8–50.3; *p* < 0.01). Personal history of solid cancer, hematologic malignancies and previous head and neck radiation therapy were not significantly associated with a higher risk of developing OSCC (Table 3).

**Table 3:** Primary and secondary risk factors for OSCC after exact matching of cases and controls

Primary factors	Cases ***n*** = 255 n (%)	Controls ***n*** = 255 n (%)	***p***-value	Odds ratio (95% CI)
**Prior personal cancer history**	24 (9.4)	31 (12.1)	0.25	
Solid cancer (excluding HNC)	16 (6.3)	29 (11.4)	0.05	0.53 (0.28–1.0)
Hematologic cancer	1 (0.4)	2 (1.2)	0.21	0.5 (0.04–5.5)
Prior HNC	7 (3.5)	0 (0.0)	0.99	N/A
**Secondary factors**				
History of dysplasia/leukoplakia	47 (18.4)	3 (1.2)	< 0.01	15.6 (4.87–50.3)
History of head and neck RT	7 (2.7)	0 (0.0)	0.99	N/A

*HNC* Head and neck cancer. *RT* Radiation therapy

## Discussion

This retrospective case-control study investigated the role of FHC, FHHNC, personal history of cancer and secondary factors in the development of OSCC. Overall, we did not find an association between FHC, FHHNC and OSCC risk, whereas a history of cancer in one or more siblings was associated with a greater OSCC risk (OR: 1.59; 95% CI: 1.17–2.89; *p* = 0.01). When secondary factors were analyzed, personal history of oral leukoplakia and dysplasia was found to be associated (15.6-times) with the development of OSCC (OR: 16; 95% CI: 4.8–50.3; *p* < 0.01).

Previous studies have reported on the role of alternative risk factors for OSCC aside from the well-documented tobacco and alcohol. *Gravello* et al. [14] in their multicenter case/control study reported a strong association between FHHNC in FDRs and oral and pharyngeal cancer (OR: 2.6, 95% CI 1.5–4.5). Specifically, patients with at least one parent affected by HNC had a 2.3-times higher risk of cancer (95% CI 1.1–4.8), whereas patients who had at least one sibling with a history of HNC were 3-times more likely to develop an oral and oropharyngeal cancer (95% CI 1.3–7.0). In addition, the risk was higher when two or more relatives were affected (OR: 2.4 95% CI 1.4–4.3). *Radoi* et al. [11] conducted a large case-control study in France evaluating FHC and personal medical history in OSCC patients. Their results showed an association between history of HNC and OSCC risk among all FDRs (OR: 1.9, 95% CI 1.2–2.8), especially in subjects aged 45 or more which showed a 2.3-times higher risk of OSCC. In 2015, the International Head and Neck Cancer Epidemiology (INHANCE) consortium conducted a large study to evaluate risk factors for HNC, including FHC and FHHNC [6]. A history of any cancer in any family member was associated with risk of HNC with young (< 45 years) and older adults (> 45 years), whereas a family history of HNC was found to be associated with older adults (> 45 years) having HNC. In a case control study, *Copper et al* [15] evaluated the role of FHHNC in FDRs in the development of head and neck malignancies; overall, FDRs (*n* = 617) of 105 patients with HNC had 31 cases of cancer of the respiratory and upper digestive tract, with 41.9% (*n* = 13) of them located in the head and neck area. Interestingly, when the frequency among all FDRs was evaluated, siblings had the highest rate of frequency (OR: 14.61 95% CI 3.1–69.1) whereas there was no evidence of association between history of cancer in parents and increased HNC risk. Similarly, in our study, a history of cancer in one or more siblings was associated with a greater OSCC risk (OR: 1.59; 95% CI: 1.17–2.89; *p* < 0.01).

Our study has several limitations including its retrospective nature and self-report of FHC/FHHNC. In addition, some providers did not systematically record FHC, which overall may have altered part of our results compared to the afore-mentioned studies (OR: 1.59 vs OR: 14.61 [10]). Finally, we did not include information about social history (family history of alcohol abuse, or heavy smoking, and environmental factors) of FDRs and SDRs, nor we considered inheritable syndromes associated with the risk of OSCC, although OSCC secondary to such conditions are rare and represent a small percentage of OSCC cases [16].

## Conclusion

In summary, our study suggests that a family history of cancer in one or more siblings may be an independent risk factor for OSCC, along with a history of previous oral leukoplakia or dysplasia. No evidence of an association was found between a family history of any cancer and increased risk of OSCC in this patient cohort from the New England region of the USA. The identification of additional risk factors for OSCC may help clinicians better identify which patient subgroups are at risk and should be screened regularly for such malignancies.

## Data Availability

The data that support the findings of this study are available from Brigham and Women’s Hospital and Dana Farber Cancer Institute, but restrictions apply to the availability of these data, which were used under license for the current study, and so are not publicly available. Data are however available from the authors upon reasonable request and with permission of Brigham and Women’s Hospital and Dana Farber Cancer Institute.
